# Development of an anti-Pfs230 monoclonal antibody as a Plasmodium falciparum gametocyte blocker

**DOI:** 10.21203/rs.3.rs-3757253/v1

**Published:** 2023-12-19

**Authors:** Emilia C. Cuccurullo, Yuemei Dong, Maria L. Simões, George Dimopoulos, Ethan Bier

**Affiliations:** Johns Hopkins University; Johns Hopkins University; Johns Hopkins University; Johns Hopkins University; University of California, San Diego

**Keywords:** Malaria, Anopheles, Plasmodium falciparum, Pfs230, gametocytes, Transmission-blocking activity, monoclonal antibody

## Abstract

Vector control is a crucial strategy for malaria elimination by preventing infection and reducing disease transmission. Most gains have been achieved through insecticide-treated nets (ITNs) and indoor residual spraying (IRS), but the emergence of insecticide resistance among *Anopheles* mosquitoes calls for new tools to be applied. Here, we present the development of a highly effective murine monoclonal antibody, targeting the N-terminal region of the *Plasmodium falciparum* gametocyte antigen Pfs230, that can decrease the infection prevalence by > 50% when fed to *Anopheles* mosquitoes with gametocytes in an artificial membrane feeding system. We used a standard mouse immunization protocol followed by protein interaction and parasite-blocking validation at three distinct stages of the monoclonal antibody development pipeline: post-immunization, post-hybridoma generation, and final validation of the monoclonal antibody. We evaluated twenty antibodies identifying one (mAb 13G9) with high Pfs230-affinity and parasite-blocking activity. This 13G9 monoclonal antibody could potentially be developed into a transmission-blocking single-chain antibody for expression in transgenic mosquitoes.

## 1. Introduction

The mosquito-transmitted protozoan parasite *Plasmodium falciparum* is the major etiological agent of human malaria, causing more than 200 million clinical cases and 500,000 deaths per year, especially in young children in sub-Saharan Africa (1).Vector control strategies such as insecticide-treated nets (ITNs) or indoor residual spraying (IRS) have been the most effective approaches for malaria control. The documented reduction in the efficacy of insecticides and anti-parasite drugs arising from the evolved resistance of mosquitoes and parasites, respectively, calls for the development of new malaria control interventions (2). The RTS, S/AS01 (RTS, S), the first-ever approved malaria vaccine, was released with a pilot program in Ghana, Kenya, and Malawi in 2019 and only demonstrates modest protective efficacy against malaria (3). During its life cycle, *Plasmodium* progresses through multiple developmental stages within the mosquito vector (sexual stage), before being transmitted to the human host through blood feeding. The injected *Plasmodium* sporozoites will migrate to the liver and invade the hepatocytes where they develop into merozoites.

The merozoites are released into the bloodstream, where they have a natural tropism for invading the red blood cells (RBCs). Within the RBC, they multiply until the cell bursts and releases merozoites that can infect other RBCs, eventually causing the clinical symptoms of malaria. It is at this point that the transition into micro-(male) and macro-(female) gametocytes occurs. The gametocytes have five distinct maturation stages: only stage V (five) gametocytes can progress through the sexual reproduction that occurs in the mosquito host (4). Once ingested by female mosquitoes with a blood meal, gametocytes undergo gametogenesis to produce male and female gametes that mate to form zygotes. The zygotes transform into the motile ookinetes which invade the mosquito midgut, all within 18–36 hours post ingestion of the infected blood meal. The ookinetes traverse the mosquito midgut epithelium and differentiate into oocysts at the midgut basal side. Upon maturation, one oocyst releases thousands of sporozoites into the mosquito’s hemolymph, eventually invading the salivary glands to complete the malaria parasite transmission cycle upon a second blood meal (5).

The complexity of the malaria parasite’s sporogonic cycle in the mosquito vector offers multiple opportunities for intervention to halt parasite transmission. Targeting parasite antigens within the mosquito serves as the basis for transmission-blocking vaccines (TBV) (6, 7) and the development of transgenic mosquitoes expressing anti-*Plasmodium* molecules (8).

While a plethora of anti-*Plasmodium* effectors have been developed to block the parasite while it invades the mosquito midgut epithelium or translocates from the midgut to the salivary glands, the molecular targets blocking the parasites at the earlier gametocyte stages remained to be fully identified (9–13). In this study, we focused on developing and producing a gametocyte-stage blocker to target the early infection stages. It has already been reported that antisera isolated from immunized mice and monoclonal antibodies targeting sexual-stage antigens could successfully inhibit *Plasmodium* infection(14–18). After gametocyte ingestion, *Plasmodium*’s sporogonic development and malaria transmission proceed by gamete fusion, achieved by species-specific male-female gamete recognition mediated by membrane proteins on their surface. According to previous studies, only three *Plasmodium* proteins have a demonstrated role in this recognition process: P48/45, P47, and P230 (19–22). Pfs230 plays a role in male/female gamete fusion, male gamete exflagellation, and interaction with erythrocytes. Due to its large size (> 230 kDa) and complex disulfide-bonded structure, recombinant expression of full-length Pfs230 has not yet been successful, however, polyclonal antisera raised against the cysteine-rich domain 1 of Pfs230 have shown *Plasmodium*-blocking activity (23). Domain 1 is relatively well conserved compared to other domains of Pfs230 and has therefore become a leading malaria transmission-blocking vaccine candidate (22, 23). Here, we used a standard immunization protocol to produce monoclonal antibodies targeting Pfs230 and identified an effective transmission-blocking clone (13G9) based on co-feeding assays with *P. falciparum* gametocyte cultures through a standard artificial membrane feeding assay (SMFA). The anti-Pfs230 monoclonal antibody 13G9 has shown the strongest anti-*Plasmodium* activity among twenty monoclonal antibody candidates tested in this study.

## 2. Results

### 2.1 Mouse-antisera generated after immunization with recombinant Pfs230 D1M domain show high reactivity *in vitro*

Pfs230 is a 230 kDa cysteine-rich protein, originally present as a 360 kDa precursor on the gametocyte surface(24). It includes 14 cysteine-rich domains (CM) and a natural protease cleavage site at position 542 (25). Previous studies have reported that high transmission-blocking activity can be achieved using the CM1 domain as an immunogenic antigen (23). In addition, analyses of polymorphisms within that region revealed only two predominant amino-acid substitutions at positions G605S and K661N, with the G605S having the highest frequency (AF 0.94) (23). A low polymorphism frequency in the targeting epitope is a desirable trait that reduces the risk of escaper mutations arising in the parasite that would impair the efficacy of the antibody. Twelve new putative missense mutations have been recently identified in the same region; however, they are based on de novo variant call data that require further validation (Fig.S3) (26).

**Figure 1:**
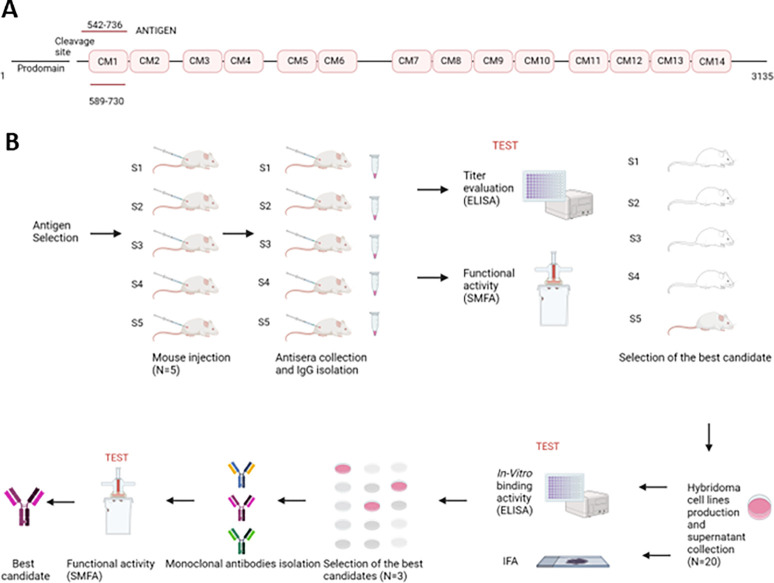
Antigen selection and general strategy. **A)** Diagram of Pfs230 protein with an outline of the prodomain, the cleavage site, the 14 cysteine-rich domains, the CM1 domain, and the region selected as the antigen. **B)** Schematic of the strategy used to obtain the mAbs targeting Pfs230 and the illustration of the *in vitro* validation assays and SMFA functional assay in the mosquitoes (created with BioRender).

**Figure 2:**
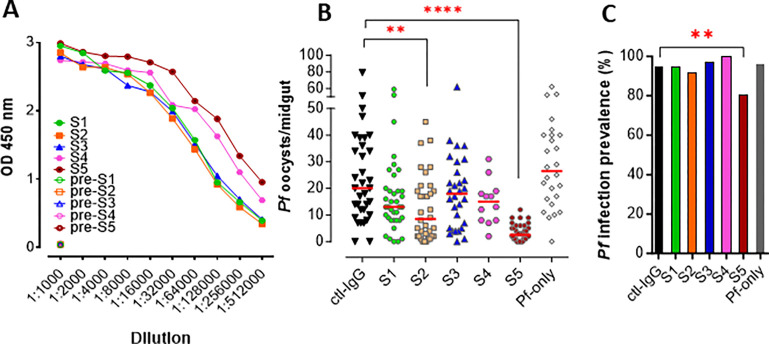
Antisera reactivity to Pfs230 *in-vitro* and analysis of functional activity. **A)** Pfs230-specific indirect-ELISA. The Pfs230 D1M domain was used as a coating antigen. The primary staining was obtained with serial dilutions of antiserum from each immunized mouse. The negative control is serum withdrawn before immunization. Secondary staining was obtained with a Peroxidase-AffiniPure Goat Anti-Mouse IgG, Fcγ Fragment Specific**. B)** and **C)** Standard membrane feeding assay (SMFA). *P. falciparum* (NF54) oocyst infection intensities **(B)**and prevalence **(C)** in midguts of laboratory-infected *Anopheles* mosquitoes 8 days post-infection (dpi). For samples from S1 to S5, the infectious blood meal was complemented with 75 μg (250 μg/ml) of IgG enriched fraction isolated from each individual antisera withdrawn from immunized mice. Ctl-IgG represents a control in which the IgG enriched fraction is isolated from pre-immunized mice. Pf- only indicates a mock control in which the infectious blood meal is complemented with PBS. All samples were adjusted to the same gametocytaemia in equal volume. Each dot represents the number of parasites in an individual gut with the median values indicated by red bars. Two-tailed p-values by the Mann-Whitney test were used to calculate the significance of infection intensity. The Fisher’s exact test was used to calculate p-value to determine the significance of infection prevalence. (*, P < 0.05; **, P < 0.01; ***, P < 0.001)

Our selected antigen for BALB/c mice immunization comprised a 195 amino acids region from the cleavage site in position 542 through the end of the cysteine-rich domain 1, which we refer to as the Pfs230 D1M domain according to previous publications (23, 26) (Fig. 1A). Test bleeds were collected after the 3rd antigen boost to assess antibody titers elicited by immunization according to standard protocols. Indirect-ELISAs (enzyme-linked immunosorbent assays) with antiserum collected from each individual mouse were used to check the antibody titrations (as illustrated in Fig. 1B). All samples were found to be reactive at a 1:512,000 dilution with mice S4 and S5 showing the highest antibody titers (Fig. 2A), confirming the highly immunogenic properties of the Pfs230 D1M domain.

### 2.2 Mouse-antisera generated by immunization with the Pfs230 D1M domain significantly reduces oocyst loads

After assessing the immunogenic response elicited by the Pfs230 D1M domain antigen, we evaluated the anti-*Plasmodium* activities of all mouse-antisera through a standard membrane-feeding assay (SMFA). Immunized mice were boosted 3 times and 50 μL of antiserum from each mouse was kept after each boosting, pooled, and used to isolate the IgG fraction. The IgG fraction from each mouse was then supplied to *Plasmodium falciparum* gametocytes cultures blood mix (with RBC and human serum) to a final concentration of 250 μg/ml and fed to the *Anopheles* female mosquitoes through a membrane feeder (Fig. 1B). The IgG fraction isolated from pre-immunized mice was used as a negative control (Fig. 2B, Ctl-IgG), together with the group of mosquitoes fed on the gametocytes blood mix supplied with PBS as the mock control (Fig. 2B, Pf-only). Since the transmission-blocking activity of previously characterized Pfs230-specific antibodies was complement-dependent (27), the human serum in the blood meal was not heat-inactivated. The infectious blood meal was delivered with high gametocytaemia to achieve a strong infection prevalence and intensity that would facilitate the selection of the most effective anti-*Plasmodium* IgGs.

The in vitro reactivities from all immunized mice (S1-S5) were comparable (Fig. 2A), however, IgGs isolated from mouse S2 and S5 showed a significantly higher level of transmission-reducing activity (Fig. 2B). Similar to previous studies describing the anti-*Plasmodium* activity of Pfs230 (28), we found a prominent reduction in oocysts number in infected mosquito midguts (8-fold reduction of median oocyst load with S5 IgG, Mann-Whitney test, p < 0.0001; and a significant reduction of infection prevalence, Fisher’s exact test, p < 0.01) (Fig. 2B, Fig. 2C). Taking these results together, we selected mouse S5 for hybridoma production to produce monoclonal antibodies.

### 2.3 Hybridoma supernatant reacts to native Pfs230

**Figure 3:**
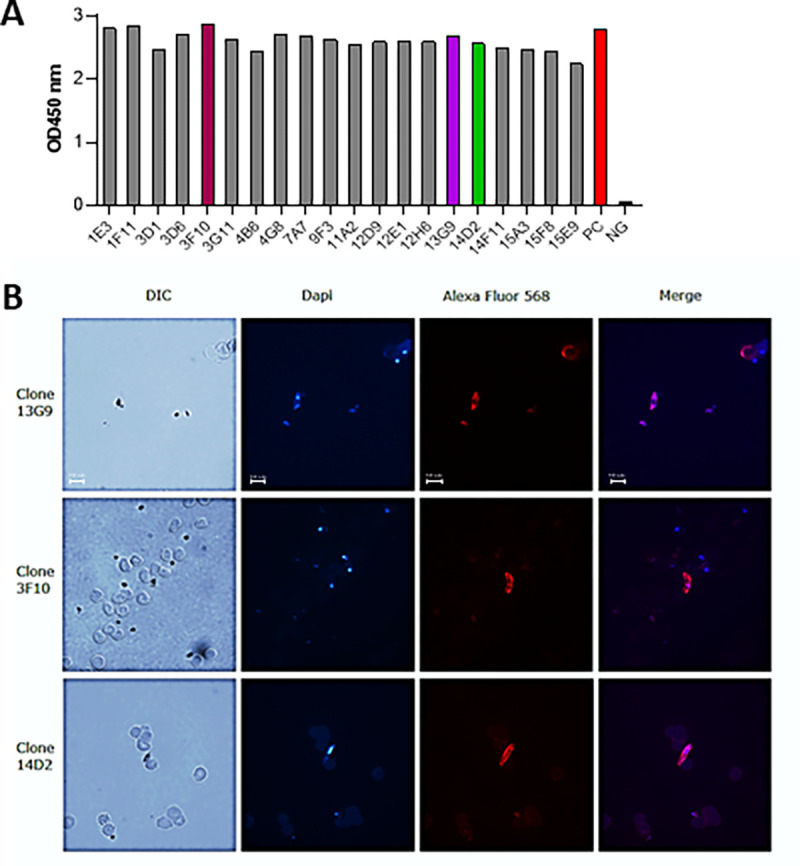
Hybridoma’s supernatant reactivity to purified and native Pfs230. **A)** Pfs230-specific indirect-ELISA. The Pfs230 D1M domain was used as a coating antigen. The primary staining was obtained with a fixed amount of supernatant from each individual hybridoma cell line. The positive control (PC in red bar) was obtained using a reactive antiserum from the experiment in Fig. 2A. The negative control (NC in black bar) is the complete media used to culture hybridoma cells. The secondary staining was obtained with a Peroxidase-AffiniPure Goat Anti-Mouse IgG, Fcγ Fragment Specific. The three colored bars were the clones selected for further analysis. **B)**Microscopy images of *P. falciparum* stage V gametocytes immunostained using supernatants from clone 13G9, 3F10, and 14D2. Detection was obtained through secondary staining with Alexa Fluor^®^ 488 anti-mouse IgG Fc Antibody. The blue spots indicate DAPI-stained gametocytes nuclei. Scale bars: 10 μm.

We employed well-established hybridoma technology to produce monoclonal antibodies. Briefly, B lymphocytes isolated from immunized mice were fused with immortal myeloma cell lines to form the hybridoma (29). B-lymphocytes isolated from mouse S5 were used to generate 20 hybridoma cell lines (1E3, 1F11, 3D1, 3D6, 3F10, 3G11, 4B6, 4G8, 7A7, 9F3, 11A2, 12D9, 12E1, 12H6, 13G9, 14D2, 14F11, 15A3, 15F8, 15E9), each expressing a monoclonal antibody targeting the Pfs230 D1M domain. To validate whether hybridoma-produced monoclonals were interacting with the Pfs230 D1M domain, we performed *in vitro* indirect-ELISA (Fig. 1B). Secondary staining with a Fcy fragment-specific peroxidase-AffiniPure goat anti-mouse IgG revealed high immunoreactivities for all 20 undiluted hybridoma supernatants, with 450 nm OD readings ranging from 2.239 to 2.874 (Fig. 3A), thereby confirming that all 20 hybridomas produce monoclonal antibodies that can bind the Pfs230 D1M domain antigen.

To assess whether the antibodies could recognize the native Pfs230 protein on the surface of *P. falciparum* NF54 gametocytes, we performed immunofluorescence assays (IFAs) (Fig. 1B) with hybridoma supernatants. Staining with a secondary Fcγ fragment-specific Peroxidase-AffiniPure Goat Anti-Mouse IgG, showed that only the supernatants collected from clones 13G9, 3F10, and 14D2 bound strongly to gametocytes (Fig. 3B), and these were thus selected for further studies to assess their parasite-blocking potential. Clones 1E3, 1F11, 3D1, 3D6, 3G11, 4B6, 4G8, 7A7, 9F3, 11A2, 12D9, 12E1, 12H6, 14F11, 15A3, 15F8 and 15E9 displayed a reactivity comparable to the negative control with unnoticeable binding of any antibodies to the gametocytes (data not shown).

### 2.4 The 13G9 monoclonal antibody has a potent *Plasmodium*-blocking activity

**Figure 4:**
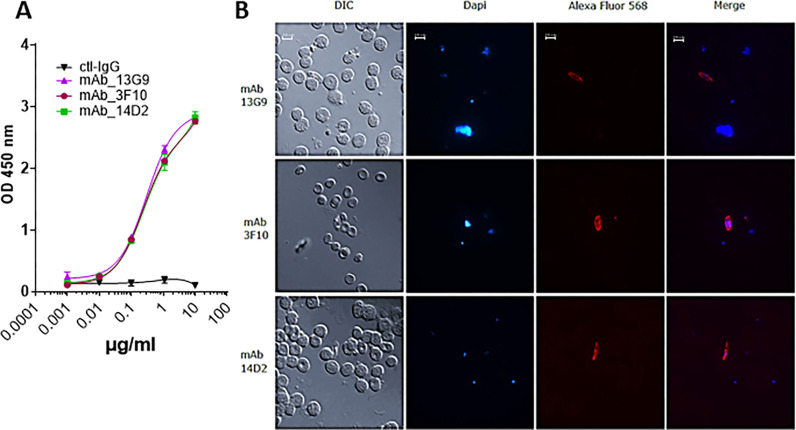
Monoclonal antibodies’ reactivity to purified and native Pfs230 in vitro. **A)** Pfs230-specific indirect-ELISA. The Pfs230 D1M domain was used as a coating antigen. The primary staining was obtained with increasing concentrations of each individual monoclonal. The negative control was obtained from the IgG enriched fraction isolated from complete hybridoma cell media. The secondary staining was obtained with Peroxidase-AffiniPure Goat Anti-Mouse IgG, Fcγ Fragment Specific **B)**Microscopy images of *P. falciparum* stage V gametocytes immunostained using 13G9, 3F10 and 14D2 monoclonals. Detection was obtained through secondary staining with Alexa Fluor^®^ 488 anti-mouse IgG Fc Antibody. The blue spots indicate DAPI-stained gametocytes nuclei. Scale bars: 10 μm.

Next, in order to assess the efficacy of candidate monoclonal antibodies to suppress *P. falciparum* infection, we first isolated IgG fractions from hybridoma’s supernatants 13G9, 3F10, 14D2 as testing groups, and IgG from complete hybridoma cell media as the negative control (Ctl-IgG) and evaluated their anti-*Plasmodium* activities by SMFA (Fig. 1B). Their reactivity to the gametocytes was again confirmed by the same assays as described above (Fig. 1B). Both *in vitro* indirect-ELISA and immunofluorescence have confirmed the specific activities of these three monoclonal antibodies (Fig. 4).

**Figure 5:**
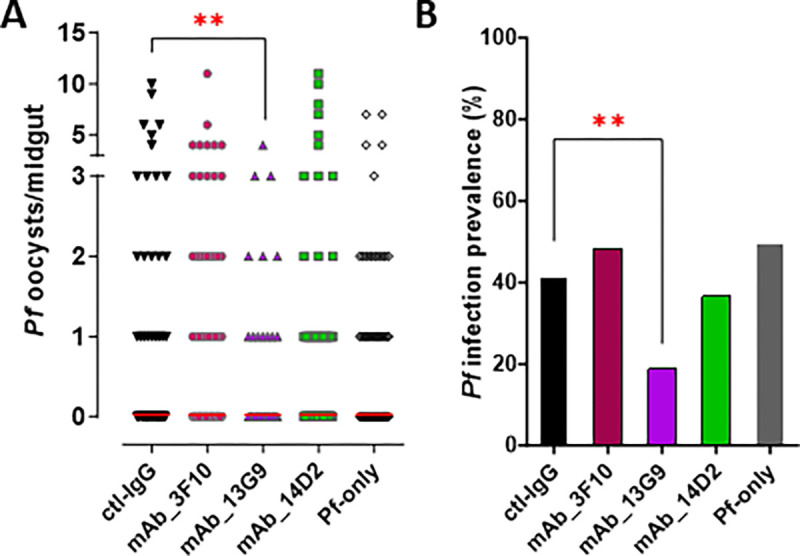
Monoclonal antibodies’ functional activity. *P. falciparum* oocyst infection intensities **(A)** and prevalence **(B)** in *Anopheles*mosquitoes at 8 dpi. For mAb samples (3F10, 13G9, and 14D2) the infectious blood meal was complemented with 30 μg of IgG (166 μg/ml) enriched fraction isolated from the supernatant of each individual hybridoma cell line. Ctl-IgGs represents a control in which the IgG enriched fraction is isolated from complete media used to culture Hybridoma cells Pf- only indicates a mock control in which the infectious blood meal is complemented with PBS. All samples were adjusted to the same gametocytaemia in equal volume. Each dot represents the number of parasites in an individual gut with the median values indicated by red bars. Two-tailed Mann-Whitney test was used to calculate the p-values to determine the significance of infection intensity. A two-tailed p-value by the Fisher’s exact test was used to calculate the significance of infection prevalence. (*, P < 0.05; **, *P* < 0.01; ***, *P* < 0.001).

Co-feeding *Anopheles* females 30 μg of 13G9 monoclonal antibody with P. falciparum gametocytes-infected blood meal (with a final concentration of 166 μg/ml) through a membrane feeder resulted in a prominent reduction of infection intensity (Fig. 5A) (2.5-fold reduction of mean oocyst load, Mann-Whitney test p = 0.0035) and infection prevalence (50% reduction, Fisher’s exact test, p = 0.0011) (Fig. 5B) at 8 days post-infection. Monoclonal antibodies 3F10 and 14D2 did not show any *Plasmodium*-blocking activity despite exhibiting binding to both the Pfs230 D1M domain and the full-length protein *in vitro* (Fig. 4), highlighting the necessity for *in vivo* functional assays in addition to the *in vitro* reactivity assays.

## 3. Discussion

Targeting the malaria parasite in the mosquito vector using transmission-blocking vaccines is a disease control strategy that has been gaining increasing interest over the past decades due to the difficulties in eliminating malaria for the lack of an effective vaccine (30). Transmission-blocking vaccine’s (TBV) mechanism of action is based on vaccination-based immunization of the human host with a parasite or mosquito antigen which is essential for *Plasmodium*’ sporogonic development in the mosquito. While such vaccines do not protect the vaccinated individual from disease, they could contribute to disease suppression at the population level since the infected individuals cannot transmit the pathogen. Three primary *P. falciparum*-encoded proteins, *Pf* s48/45, Pf s230, and *Pf* s25 are currently considered lead candidates for TBV development. *Pf*s230 is a member of the six-cysteine (6-Cys) family and is composed of fourteen 6-Cys domains forming a complex intra-domain disulfide bond structure, the site of recognition for antibodies binding to conformational epitopes. Accordingly, polyclonal antibodies raised through immunization with the whole gametocyte did not recognize the antigen in its reduced form (27). Previous studies aiming to identify the most suitable region of Pfs230 to use as an antigen showed that the N-terminal Prodomain, which lacks CM cysteine-rich domains, could also achieve this goal, suggesting that Transmission-blocking antibodies may also be directed against non-conformational epitopes (31, 32)

Extending these prior studies focused on the research of the best antigen to select and utilize in a vaccine development context (33, 34), here we directed our attention to generating and isolating an effective monoclonal antibody targeting the early stages of parasite development within the mosquito, using the Pfs230 D1M domain previously demonstrated to elicit strong immuno-response (33, 34). We introduced one additional level of testing to the standard pipeline of monoclonal antibody production by selecting the monoclonal antibody that displayed the highest functional parasite-blocking activity.

In a cohort of 5 mice immunized with the Pfs230 D1M domain in identical conditions, the IgG fraction isolated from the mouse S5 led to a significantly lower oocyst count and prevalence when fed with an infectious meal to *Anopheles* mosquitoes, confirming his selection as the best candidate in terms of both potency and effectiveness of the immune response elicited.

Alternatively to transmission blocking vaccines, new therapeutic approaches developed for malaria prevention and therapy include the use of recombinant transmission-blocking antibodies. The ability to target antigens expressed in the early sexual stages potentially allows to reduce the infection intensity within the mosquito host. Cocktails of different antibodies or bispecific molecules could serve this specific purpose and render this strategy more effective and long-lasting (35). In the recent past, panels of monoclonal antibodies targeting Pfs230 with a different range of affinity have been developed by various research groups, confirming the need to generate new reagents towards this antigen (36).

Finally, the same monoclonal antibodies developed as transmission-blocking molecules could be used in the context of malaria eradication strategies based on population modification of the vector host. Since Pfs230’s essential biological function for malaria transmission takes place in the mosquito midgut lumen after ingestion of gametocytes through an infected blood meal, the 13G9 monoclonal antibody could potentially be developed into a single-chain antibody that could be expressed and secreted into the midgut lumen through an appropriate promoter.

Transgenic mosquitoes expressing transmission-blocking molecules and able to transmit the desired traits with a super mendelian inheritance are already a reality and proven to reduce the parasite burden below the transmission level in cage trial experimental settings (37).

A combination of multiple effectors targeting Plasmodium at different developmental stages is most likely the most effective strategy to overcome the insurgence of parasite resistance due to selective pressure during host-pathogen coevolution (38). In this light, the generation and validation of a new transmission-blocking agent targeting the early stage of the parasite in addition to already established ones is useful for the goal of malaria eradication.

## 4. Materials and methods

### 4.1 Antigen production, immunization, and monoclonal antibody production

The Pfs230 D1M domain (SVLQSGALPSVGVDELDKIDLSYETTESGDTAVSEDSYDKYASQNTNKEYVCDFTDQLKPTESGPKVKKCEVKVNEPLIKVKIICPLKGSVEKLYDNIEYVPKKSPYVVLTKEETKLKEKLLSKLIYGLLISPTVNEKENNFKEGVIEFTLPPVVHKATVFYFICDNSKTEDDNKKGNRGIVEVYVEPYGNKING) was codon optimized according to the expression in *E. coli* (Fig. S1). The synthesized sequence with a 6x HIS tag on the C-terminus was cloned into a pET30a vector (EDM Millipore) and expressed in *E. coli* BL21 Star (DE3). Induction of recombinant protein was achieved with IPTG at 15°C for 16 hours as per standard protocols (GenScript). Cell pellets were resuspended with lysis buffer followed by sonication. The supernatant resulting from centrifugation was kept for future purification. Target proteins were dialyzed and sterilized by a 0.22μm filter before being stored in aliquots. The concentration was determined by BCA protein assay with BSA as a standard. The protein purity and molecular weight were determined by standard SDS-PAGE along with western blot confirmation (GenScript, Fig. S2).

Five mice were immunized according to standard immunization protocol (39) and 50 μl of antiserum was collected after every injection. Hybridoma cell lines (N = 20: 1E3, 1F11, 3D1, 3D6, 3F10, 3G11, 4B6, 4G8, 7A7, 9F3, 11A2, 12D9, 12E1, 12H6, 13G9,14D2, 14F11, 15A3) were produced by fusion of mouse myeloma cells SP2/0 with splenocytes from BALB/c immunized mouse S5 as for standard protocol (GenScript).

### 4.2 Cell lines

Hybridoma cells were thawed into a 37 °C water bath, adapted to culturing conditions, and kept in complete medium (90% DMEM + 10% FBS, Gibco), 5% CO2, 37 °C.

### 4.3 IgGs and monoclonal antibody isolation

IgG fractions from antisera and monoclonal antibodies from hybridoma supernatants clone 13G9, 3F10, or 14D2 were isolated using NAb^™^ Protein G kit (Thermo Scientific^™^) and stored in Phosphate Buffered Saline, pH 7.4 after buffer exchange with Zeba Desalt Spin columns 4 MWCO (Thermo Scientific^™^). Antibody stocks were concentrated at the desired volume with an Amicon^®^ ultra – centrifugal filter unit (30kDa filter Millipore-Sigma) and stored at −20 °C.

### 4.4 ELISAs (enzyme-linked immunosorbent assay)

The *in vitro* binding activity of antisera, hybridoma supernatants, and monoclonals was evaluated by ELISA. 96 well microtiter plates (Immulon 4HBX - Thermo Scientific^™^) were coated with 1μg/ml of the Pfs230 D1M domain in Carbonate –Bicarbonate buffer pH 9.6 and kept overnight at 4°C in a humidified chamber. Unbound target protein was removed by four rinses with PBST buffer and wells were blocked for 1 hr at room temperature (RT) with superblock blocking buffer PBS (Thermo Scientific^™^). Following three rinses with PBS tween 20 0.01% buffer (PBST), plates were incubated overnight in a humidified chamber with serial dilutions of antiserum from each immunized or naïve mouse, 100 μl of Hybridoma supernatant, or increasing concentrations of 13G9, 3F10, or 14D2 monoclonal antibodies in superblock blocking buffer PBS under gentle rocking. Wells lacking primary antibodies were used as a negative control. Following four rinses with PBST, wells were incubated for 1 hr at RT with 100 μL of peroxidase-affinipure goat anti-mouse IgG, Fcy fragment specific secondary antibody (Jackson Immunoresearch Laboratories) diluted 1:5000 in superblock blocking buffer PBS (Thermo Scientific^™^). Following four rinses with PBST buffer, wells were incubated with TMB substrate (Sera care) at room temperature in the dark with gentle rocking. The reaction was stopped after 20 minutes with 50 μl of stop solution (Thermo Scientific^™^) per well. Absorbance reads were immediately taken in duplicates at 450 nm with a microplate reader (Azure). Each sample was tested in at least 2 independent experiments.

### 4.5 Immunohistochemical staining and microscopy

Blood smears of the *P. falciparum* gametocytes were air-dried after methanol fixation and evaluated by IFA. After membrane permeabilization and blocking of nonspecific binding (3% BSA, 0.1% saponin in PBS for 1hr at RT) fallowed by 3 PBS washes, the preparations were then incubated individually with the hybridoma supernatants, or 10 μg/mL monoclonal antibodies from 13G9, 3F10, 14D2 clones, or IgG enriched fraction isolated from complete Hybridoma cell media in 1%BSA in PBS at room temperature for 1 hr. AlexaFluor ^™^ 488 anti-mouse IgG Fc antibody (1:1000 dilution; Invitrogen) in PBS was used for secondary staining and detection. After 3 washes with PBS, slides were let dry and mounted with Prolong^™^ Gold Antifade (Invitrogen). Microscopic examination was performed the fallowing day with a Zeiss AXIO fluorescence microscope system. Each sample was tested in at least 2 independent experiments.

### 4.6 Mosquito rearing, *Plasmodium falciparum* infection and statistical analysis

*Anopheles mosquitoes* were maintained on a 10% sugar solution at 27°C and 70%–80% humidity and a 12-hours light/dark cycle according to standard rearing procedures. Anti-*Plasmodium* activity was determined by SMFA. The infectious blood meal was prepared with NF54 *P. falciparum* gametocyte cultures, active serum, and RBCs (provided by the Hopkins Malaria Research Institute Core Facility) (40, 41) complemented with our experimental samples (IgG fractions from antisera or hybridoma supernatants) or PBS. After starving the adult mosquitoes for 3 to 6 hours, they were allowed to feed for 1hr on artificial membrane feeders at 37°C. Only the cohort of fully engorged blood-fed mosquitoes was selected and kept until 8 dpi for oocyst counting and infection prevalence assessment. Midguts were dissected out in phosphate-buffered saline (PBS) and stained in 0.02% PBS–buffered mercurochrome (Millipore Sigma). Oocysts were examined using a light-contrast microscope (Olympus). All experiments were repeated at least 2 times. Each biological replicate corresponds to a different mosquito population cage, and each population corresponds to a different generation. All graphs were generated using GraphPad Prism8 software, and the statistical methods used for each experiment are indicated in the respective figure legends.

## References

[1] World Health Organization. World malaria report 2022 [Internet]. Available from: https://www.who.int/teams/global-malaria-programme

[2] World Health Organization. World malaria report 2020 – 20 years of global progress & challenges.

[3] Chandramohan D, Zongo I, Sagara I, Cairns M, Yerbanga RS, Diarra M, Seasonal Malaria Vaccination with or without Seasonal Malaria Chemoprevention. New England Journal of Medicine. 2021 Sep 9;385(11):1005–17. 34432975 10.1056/NEJMoa2026330

[4] Gardiner DL, Trenholme KR. Plasmodium falciparum gametocytes: Playing hide and seek. Vol. 3, Annals of Translational Medicine. AME Publishing Company; 2015. 10.3978/j.issn.2305-5839.2015.01.23 PMC4381475 25861600

[5] Venugopal K, Hentzschel F, Valkiūnas G, Marti M. Plasmodium asexual growth and sexual development in the haematopoietic niche of the host. Vol. 18, Nature Reviews Microbiology. Nature Research; 2020. p. 177–89. 10.1038/s41579-019-0306-2 PMC7223625 31919479

[6] Carter R, Mendis KN, Miller LH, Molineaux L, Saul A. Malaria transmission-blocking vaccines - how can their development be supported. Nat Med. 2000;6(3). 10.1038/73062 10700212

[7] Carter R. Transmission blocking malaria vaccines. Vaccine [Internet]. 2001;19(17):2309–14. Available from: https://www.sciencedirect.com/science/article/pii/S0264410X00005211 11257353 10.1016/s0264-410x(00)00521-1

[8] Dong S, Dong Y, Simões ML, Dimopoulos G. Mosquito transgenesis for malaria control. Vol. 38, Trends in Parasitology. Elsevier Ltd; 2022. p. 54–66. 10.1016/j.pt.2021.08.001 34483052

[9] Barr PJ, Green KM, Gibson HL, Bathurst IC, Quakyi IA, Kaslow DC. Recombinant Pfs25 Protein of Plasmoch’um falciparum Elicits Malaria Transmission-blocking Immunity in Experimental Animals [Internet]. Available from: http://rupress.org/jem/article-pdf/174/5/1203/1672511/1203.pdf 10.1084/jem.174.5.1203 PMC2118997 1940798

[10] McLeod B, Miura K, Scally SW, Bosch A, Nguyen N, Shin H, Potent antibody lineage against malaria transmission elicited by human vaccination with Pfs25. Nat Commun. 2019 Dec 1;10(1). 10.1038/s41467-019-11980-6 PMC6760140 31551421

[11] Scally SW, McLeod B, Bosch A, Miura K, Liang Q, Carroll S, Molecular definition of multiple sites of antibody inhibition of malaria transmission-blocking vaccine antigen Pfs25. Nat Commun. 2017 Dec 1;8(1). 10.1038/s41467-017-01924-3 PMC5691035 29146922

[12] Burkot TR, Da ZW, Geysen HM, Wirtz RA, Saul A. Fine specificities of monoclonal antibodies against the Plasmodium falciparum circumsporozoite protein: recognition of both repetitive and non-repetitive regions. Parasite Immunol. 1991;13(2):161–70. 2052404 10.1111/j.1365-3024.1991.tb00272.x

[13] Oludada OE, Costa G, Burn Aschner C, Obraztsova AS, Prieto K, Canetta C, Molecular and functional properties of human Plasmodium falciparum CSP C-terminus antibodies. EMBO Mol Med. 2023 Jun 7;15(6). 10.15252/emmm.202317454 PMC10245032 37082831

[14] Theisen M, Jore MM, Sauerwein R. Towards clinical development of a Pfs48/45-based transmission blocking malaria vaccine. Vol. 16, Expert Review of Vaccines. Taylor and Francis Ltd; 2017. p. 329–36. 10.1080/14760584.2017.1276833 28043178

[15] Kundu P, Semesi A, Jore MM, Morin MJ, Price VL, Liang A, Structural delineation of potent transmission-blocking epitope I on malaria antigen Pfs48/45. Nat Commun. 2018 Dec 1;9(1). 10.1038/s41467-018-06742-9 PMC6203815 30367064

[16] Coelho CH, Tang WK, Burkhardt M, Galson JD, Muratova O, Salinas ND, A human monoclonal antibody blocks malaria transmission and defines a highly conserved neutralizing epitope on gametes. Nat Commun. 2021 Dec 1;12(1). 10.1038/s41467-021-21955-1 PMC7979743 33741942

[17] Canepa GE, Molina-Cruz A, Yenkoidiok-Douti L, Calvo E, Williams AE, Burkhardt M, Antibody targeting of a specific region of Pfs47 blocks Plasmodium falciparum malaria transmission. NPJ Vaccines. 2018 Dec 1;3(1). 10.1038/s41541-018-0065-5 PMC6039440 30002917

[18] Yenkoidiok-Douti L, Williams AE, Canepa GE, Molina-Cruz A, Barillas-Mury C. Engineering a Virus-Like Particle as an Antigenic Platform for a Pfs47-Targeted Malaria Transmission-Blocking Vaccine. Sci Rep. 2019 Dec 1;9(1). 10.1038/s41598-019-53208-z PMC6856133 31727945

[19] Van Dijk MR, Janse CJ, Thompson J, Waters AP, Braks JAM, Dodemont HJ, A Central Role for P48/45 in Malaria Parasite Male Gamete Fertility The central role of zygote formation in the life cycle and transmission of the parasite makes gametes and. Vol. 104, Cell. 2001. 10.1016/s0092-8674(01)00199-4 11163248

[20] Khan SM, Franke-Fayard B, Mair GR, Lasonder E, Janse CJ, Mann M, Proteome analysis of separated male and female gametocytes reveals novel sex-specific Plasmodium biology. Cell. 2005 Jun 3;121(5):675–87. 15935755 10.1016/j.cell.2005.03.027

[21] van Schaijk BCL, van Dijk MR, van de Vegte-Bolmer M, van Gemert GJ, van Dooren MW, Eksi S, Pfs47, paralog of the male fertility factor Pfs48/45, is a female specific surface protein in Plasmodium falciparum. Mol Biochem Parasitol. 2006 Oct;149(2):216–22. 16824624 10.1016/j.molbiopara.2006.05.015

[22] Eksi S, Czesny B, Van Gemert GJ, Sauerwein RW, Eling W, Williamson KC. Malaria transmission-blocking antigen, Pfs230, mediates human red blood cell binding to exflagellating male parasites and oocyst production. Mol Microbiol. 2006 Aug;61(4):991–8. 16879650 10.1111/j.1365-2958.2006.05284.x

[23] MacDonald NJ, Nguyen V, Shimp R, Reiter K, Herrera R, Burkhardt M, Structural and immunological characterization of recombinant 6-cysteine domains of the plasmodium falciparum sexual stage protein Pfs230. Journal of Biological Chemistry. 2016 Sep 16;291(38):19913–22. 27432885 10.1074/jbc.M116.732305 PMC5025679

[24] Brooks SR, Williamson KC. Proteolysis of Plasmodium falciparum surface antigen, Pfs230, during gametogenesis [Internet]. Vol. 106, Molecular and Biochemical Parasitology. 2000. Available from: www.elsevier.com/locate/parasitology 10.1016/s0166-6851(99)00201-7 10743612

[25] Williamson KC, Keister DB, Muratova O, Kaslow DC. Recombinant Pfs230, a Plasmodium falciparum gametocyte protein, induces antisera that reduce the infectivity of Plasmodium falciparum to mosquitoes. Mol Biochem Parasitol. 1995 Dec;75(1):33–42. 8720173 10.1016/0166-6851(95)02507-3

[26] Singh K, Burkhardt M, Nakuchima S, Herrera R, Muratova O, Gittis AG, Structure and function of a malaria transmission blocking vaccine targeting Pfs230 and Pfs230-Pfs48/45 proteins. Commun Biol. 2020 Dec 1;3(1). 10.1038/s42003-020-01123-9 PMC7381611 32709983

[27] Read D, Lensen AH, Begarnie S, Haley S, Raza A, Carter R. Transmission-blocking antibodies against multiple, non-variant target epitopes of the Plasmodium fakiparum gamete surface antigen Pfs230 are all complement-fixing. Parasite Immunol. 1994; 10.1111/j.1365-3024.1994.tb00305.x 7532850

[28] Miura K, Takashima E, Deng B, Tullo G, Diouf A, Moretz SE, Functional comparison of plasmodium falciparum transmission-blocking vaccine candidates by the standard membrane-feeding assay. Infect Immun. 2013;81(12):4377–82. 24042109 10.1128/IAI.01056-13 PMC3838000

[29] Holzlöhner P, Hanack K. Generation of murine monoclonal antibodies by hybridoma technology. Journal of Visualized Experiments. 2017 Jan 2;2017(119). 10.3791/54832 PMC5407676 28117810

[30] Nikolaeva D, Draper SJ, Biswas S. Toward the development of effective transmission-blocking vaccines for malaria. Vol. 14, Expert Review of Vaccines. Expert Reviews Ltd.; 2015. p. 653–80. 10.1586/14760584.2015.993383 25597923

[31] Tachibana M, Wu Y, Iriko H, Muratova O, MacDonald NJ, Sattabongkot J, N-terminal prodomain of Pfs230 synthesized using a cell-free system is sufficient to induce complement-dependent malaria transmission-blocking activity. Clinical and Vaccine Immunology. 2011 Aug;18(8):1343–50. 21715579 10.1128/CVI.05104-11 PMC3147338

[32] Singh SK, Thrane S, Chourasia BK, Teelen K, Graumans W, Stoter R, Pfs230 and Pfs48/45 fusion proteins elicit strong transmission-blocking antibody responses against plasmodium falciparum. Front Immunol. 2019;10(JUN). 10.3389/fimmu.2019.01256 PMC6560166 31231386

[33] Healy SA, Anderson C, Swihart BJ, Mwakingwe A, Gabriel EE, Decederfelt H, Pfs230 yields higher malaria transmission-blocking vaccine activity than Pfs25 in humans but not mice. Journal of Clinical Investigation. 2021 Apr 1;131(7). 10.1172/JCI146221 PMC8011888 33561016

[34] Salinas ND, Ma R, Dickey TH, McAleese H, Ouahes T, Long CA, A potent and durable malaria transmission-blocking vaccine designed from a single-component 60-copy Pfs230D1 nanoparticle. NPJ Vaccines [Internet]. 2023 Aug 18;8(1):124. Available from: https://www.nature.com/articles/s41541-023-00709-8 37596283 10.1038/s41541-023-00709-8 PMC10439124

[35] Coelho CH, Jore MM, Canepa GE, Barillas-Mury C, Bousema T, Duffy PE. Antibody Therapy Goes to Insects: Monoclonal Antibodies Can Block Plasmodium Transmission to Mosquitoes. Vol. 36, Trends in Parasitology. Elsevier Ltd; 2020. p. 880–3. 10.1016/j.pt.2020.08.009 33036937

[36] Lyons FMT, Gabriela M, Tham WH, Dietrich MH. Plasmodium 6-Cysteine Proteins: Functional Diversity, Transmission-Blocking Antibodies and Structural Scaffolds. Vol. 12, Frontiers in Cellular and Infection Microbiology. Frontiers Media S.A.; 2022. 10.3389/fcimb.2022.945924 PMC9309271 35899047

[37] Carballar-Lejarazú R, Dong Y, Pham TB, Tushar T, Corder RM, Mondal A, Dual effector population modification gene-drive strains of the African malaria mosquitoes, Anopheles gambiae and Anopheles coluzzii. Proceedings of the National Academy of Sciences [Internet]. 2023 Jul 18;120(29). Available from: 10.1073/pnas.2221118120 PMC10629562 37428915

[38] Dong Y, Simões ML, Dimopoulos G. Versatile transgenic multistage effector-gene combinations for Plasmodium falciparum suppression in Anopheles [Internet]. 2020. Available from: https://www.science.org 10.1126/sciadv.aay5898 PMC7220273 32426491

[39] Greenfield EA. Standard Immunization of Mice, Rats, and Hamsters. Cold Spring Harb Protoc. 2020;2020(3):82–4. 10.1101/pdb.prot100297 32123014

[40] Dong Y, Das S, Cirimotich C, Souza-Neto JA, McLean KJ, Dimopoulos G. Engineered anopheles immunity to plasmodium infection. PLoS Pathog. 2011 Dec;7(12). 10.1371/journal.ppat.1002458 PMC3245315 22216006

[41] Lobo NF, Clayton JR, Fraser MJ, Kafatos FC, Collins FH. High efficiency germ-line transformation of mosquitoes. Nat Protoc. 2006 Aug;1(3):1312–7. 17406416 10.1038/nprot.2006.221

